# Detecting Misleading Information on COVID-19

**DOI:** 10.1109/ACCESS.2020.3022867

**Published:** 2020-09-09

**Authors:** Mohamed K. Elhadad, Kin Fun Li, Fayez Gebali

**Affiliations:** Department of Electrical and Computer EngineeringUniversity of Victoria 8205 Victoria V8W 2Y2 Canada

**Keywords:** Coronavirus, COVID-19, fake news detection, infodemic, misleading information, pandemic, SARS-CoV-2, social media, social networks, text classification, text mining, web mining, WHO

## Abstract

This article addresses the problem of detecting misleading information related to COVID-19. We propose a misleading-information detection model that relies on the World Health Organization, UNICEF, and the United Nations as sources of information, as well as epidemiological material collected from a range of fact-checking websites. Obtaining data from reliable sources should assure their validity. We use this collected ground-truth data to build a detection system that uses machine learning to identify misleading information. Ten machine learning algorithms, with seven feature extraction techniques, are used to construct a voting ensemble machine learning classifier. We perform 5-fold cross-validation to check the validity of the collected data and report the evaluation of twelve performance metrics. The evaluation results indicate the quality and validity of the collected ground-truth data and their effectiveness in constructing models to detect misleading information.

## I. Introduction

At the end of December 2019, the World Health Organization (WHO) was informed of a cluster of pneumonia cases of unknown cause that were detected in the city of Wuhan, Hubei Province, China. Initially, these patients were diagnosed as having acute pneumonia. Most of them worked in a wet market in Wuhan and showed common symptoms of fever, dry cough, tiredness, and in more severe cases breathing difficulty. However, these symptoms were not of acute pneumonia as was first thought. With the increasing number of cases, China informed the WHO of the situation and its unknown cause in early January 2020 [1].

The WHO named the virus “Severe Acute Respiratory Syndrome Coronavirus 2 (SARS-CoV-2)” and the disease as “Coronavirus Disease (COVID-19)”. COVID-19 is a global health problem that requires extreme caution, strict maintenance of personal and general hygiene, and the cleanliness of all places. These practices help in avoiding the occurrence of mutations so that the virus can be controlled and contained. All reports issued by the WHO indicate that the epidemiological situation (since the beginning of January 2020) is very critical and scientists are frantically working to develop a vaccine to eradicate the virus. An effective vaccine is expected to be available to the public between December 2020 and June 2021 [2].

Transportation means and social network platforms render the world a small village. As far as transportation is concerned, it has become easy to transport people from one place to another. This promotes the circulation of COVID-19 very quickly and makes it a pandemic [2]. As for social network platforms, they play a vital and effective role not only in spreading misleading information related to COVID-19 but in all matters of our daily lives as well as the various crises and conflicts around the world. With the presence of a new virus whose characteristics and details are not fully known yet, and with a state of fear and panic among the general public, the spread and circulation of misleading information about this virus and its impact are ubiquitous.

The misleading information may be intended to disrupt the economy of countries, reduce people’s confidence in their governments, or promote a specific product to achieve enormous profits. This has already happened with COVID-19. The shared misleading information about lockdowns, vaccinations, and death statistic, have fueled the panic of purchasing groceries, sanitizers, masks, and paper products. This led to shortages that disrupted the supply chain and exacerbated demand-supply gaps and food insecurity. Moreover, it has caused a sharp decline in the international economy, severe losses in the value of crude oil, and the collapse of the world’s stock markets [3]–[5]. Additionally, some people have lost faith in their governments as in Italy and Iran, due to the spread of COVID-19 and the shortage of medical protection products all over the world [6], [7]. All these are leading the world into an economic recession [5], [8], and [9].

The WHO has issued numerous data, directives, and warnings that are not only related to COVID-19 but also the “Infodemic” [10]. Infodemic is like a disease that spreads and circulates in the form of misleading information. It is very challenging to verify the validity, credibility, and correctness of the shared information, especially if it is related to a horrific disease that is a threat to humanity [11]. The WHO has asked popular search engines, such as Google, Yahoo, and Bing, and many social network platforms to display its officially issued reports and information as top hits of any search that is related to COVID-19 [12]. It is evident from this WHO request that utmost care and caution must be exercised when selecting sources of information. We should not rely on what is promoted on social networks but rather on reliable and unbiased information sources such as the WHO, global scientific research bodies, and NGOs. Hence, there is an urgent need to provide a tool for the public to verify the trustworthiness of information related to COVID-19 [1].

In this article, we introduce a model to detect misleading information in the English language, with the COVID-19 pandemic as our case study. For our ground-truth data, we decided to gather COVID-19 related information from international, and what we perceived as reliable and unbiased, institutions. We also collected facts from different fact-checking websites in addition to the information found in official reports and news related to the pandemic from the WHO, UNICEF, and the UN official websites [1], [13], [14]. Our detection process is based on the ensembled learning of ten machine learning classifiers that are built on the collected ground-truth data.

Section II presents the related work of misleading-information detection. Section III introduces the proposed misleading-information detection system, with the details of the 4-stage process. The experimental setup and results are discussed in Section IV, while Section V concludes and suggests directions for future work. It is worth noting that, in this article, we use the terms ***misleading*** and ***fake*** interchangeably.

## II. Related Work

Most of the misleading-information detection systems deploy machine learning techniques to help users in classifying whether the data they are viewing is misleading or not. This classification is done by comparing the given data with some pre-known corpora that contain both misleading and deemed truthful information [15], [16].

For deploying machine learning techniques in building misleading-information detection models, all training data should pass through these stages: data preparation and preprocessing, feature engineering (feature selection and feature extraction), and model selection and building. These typical stages facilitate the handling of the large amount of data needed in building a detection model [17], [18]. Many available misleading-information detection websites could be used to search for pre-checked data (e.g., Snopes.com, PolitiFact.com, Factcheck.org, etc.). However, these websites are mostly human-based, where the analysis of data is carried out manually. This analysis is performed by expert analysts who are intimately familiar with the subject context. The manual approach is slow, expensive, highly subjective, biased, and has become impractical due to the huge volume of available data on social networks [19]–[21]. Hence, the process of automated classification of data represents an exciting and productive area of study.

To date, many automated misleading-information [22] detection systems have been proposed. Kaliyar and Singh provided a comprehensive survey of the detection of misleading information on various social network platforms. Zhang and Ghorbani [23] presented a comprehensive overview of the recent findings related to fake news. Moreover, they characterized the impact of online fake news, presented state-of-the-art detection methods, and discussed the commonly used datasets employed to build models to classify fake news. In the same context, Collins and Erascu [24] also gave an overview of the various models in detecting fake news and the different types of fake news. They found the techniques that combine humans and machines bring very satisfactory results when compared to systems that depend only on either one of them.

Al Asaad *et al.* [25] proposed a news credibility verification model that combines several machine learning techniques for text classification. They tested the effectiveness of their model on a fake/real news dataset using Multinomial Naïve Bayes and Lagrangian Support Vector Machine classification algorithms. Nakamura *et al.* [26] proposed a hybrid fake news detection system that employs the multinomial voting algorithm. They tested their system with multiple fake news datasets, using five machine learning algorithms: Naïve Bayes, Random Forest, Decision Tree, Support Vector Machine, and k-Nearest Neighbors.

Ibrishimova and Li [27] studied various definitions of fake news and proposed a definition based on absolute factual accuracy and relative reliability of the source. Moreover, they proposed a fake news detection framework, which utilizes both manual and automated knowledge verification and stylistic features. Elhadad *et al.* [16] proposed a model for detecting fake news on social network platforms. They selected hybrid features from metadata of the news documents to build a feature vector for the detection task. They tested the effectiveness of their technique using nine machine learning algorithms on three datasets. Shu *et al.* [28] introduced a framework for fake news collection, detection, and visualization. They collected fake and real news articles from fact-checking websites and related social interaction from social media. Then they extracted news and social interaction features to build various fake news detection models. Finally, they presented a visualization tool for the detected fake news data.

Posadas-Durán *et al.* [29] introduced a new resource to detect misinformation from news websites. They collected a dataset of news in the Spanish language extracted from several websites. The corpus data have been labeled as fake or real for fake news detection. Also, each instance of the dataset has a news category assigned to it: Science, Sport, Education, Economy, Politics, Entertainment, Health, Security, or Society. Moreover, they introduced a style-based fake news detection method. They tested their method using four machine learning algorithms (Support Vector Machine with linear kernel, Logistic Regression, Random Forest, and XGBoosting) on their proposed dataset.

Wang [30] introduced a new dataset for fake news. The dataset consists of 12.8K manually labeled short statements in various contexts from PolitiFact.com. Notably, this dataset was regarded as the first large dataset related to fake news detection. It is an order of magnitude larger than previous public fake news datasets. Thorne *et al.* [31] proposed yet another dataset that has been collected from Wikipedia pages. This dataset mainly serves applications related to stance detection. It contains around 185K documents with true-text, non-news types of articles from Wikipedia pages, while the fake news portion is collected from crowdsourcing.

Shu *et al.* [32] introduced a fake news data repository “FakeNewsNet”. FakeNewsNet contains two comprehensive datasets with diverse features in news content, social context, and spatiotemporal information. Asr and Taboada [33] reviewed the available misinformation detection datasets and introduced the “MisInfoText” repository to address the lack of datasets with reliable labels. MisInfoText repository contains three data categories: links to all the publicly available textual fake news datasets, features to collect data directly from fact-checking websites, and datasets originally published in [30], [31], [34]–[39].

In summary, many existing works focus on building misleading-information detection systems. Most of them rely on manually labeled data for the detection process. In this article, we introduce a technique to build misleading-information detecting systems, by using ground-truth collected from reliable and unbiased (at least from the developer’s perspective) information sources. Presently, we focus on data related to the COVID-19 outbreak.

## III. The Proposed Detection Model

**FIGURE 1.**
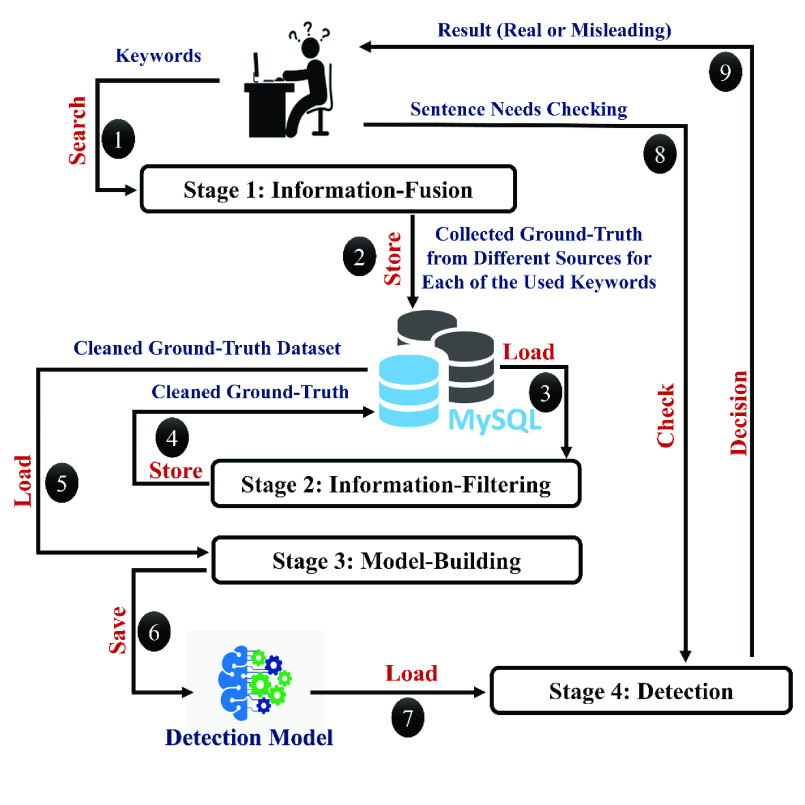
The proposed misleading-information detection framework.

To build a detection system for the pandemic news, we must first decide on how to judge COVID-19 related information, and what are the sources that we can rely on for evaluating each data instance. Fig. 1 shows the block diagram of our proposed misleading-information detection framework. The process for detecting misleading information is divided into four main stages: Information-Fusion, Information-Filtering, Model-Building, and Detection.

### A. Information-Fusion Stage

The accuracy of any detection system is highly affected by the quality of data used in building the detection model, the machine learning algorithm employed, and how these data describe the facts related to the topic of interest. Hence, when the topic of interest is critical, it is essential to ensure the accuracy and reliability of information sources and not to be drawn into shared information and news from unreliable entities. Therefore, reliance on perceptions and feelings should be avoided.

We must rely only on documented information and facts without making any modifications. As the COVID-19 pandemic is more than a purely medical event and is of concern to all people, it is necessary to depend on reliable and authoritative sources to get our information. With more scrutiny, we should be able to find medical and other organizations that try not to spread fear and terror to the public. Moreover, they should be impartial and objective in their handling of information and news of the COVID-19 outbreak crisis.

For all the previously mentioned reasons, we decided to get our COVID-19 ground-truth mainly by scraping the websites of the WHO and its regional branches, as well as UNICEF [13] and its affiliated bodies, and of course the UN [40], [41]. We extracted all the information related to COVID-19 outbreak from these organizations’ daily situation reports [2] [42], the briefing of the WHO Director-General on COVID-19 [43], in addition to the news published on their websites’ newsroom [44].

Moreover, we utilized the Google Fact Check Tools API [45], which allows users to browse and search for facts from different fact-checking websites around the world including:

– *opensecrets.org*
– *snopes.com*
– *factcheck.afp.com*
– *www.washingtonpost.com/news/fact-checker*
– *factcheck.org*
– *politifact.com*

We did not employ information published by the official accounts of the health ministries in various countries or any of the organizations and research centers affiliated with a single country. Rather, there is a reliance on international organizations to avoid biased and inaccurate statements and information.

For querying the fact-checking websites, we used the following search keywords which are related to the coronavirus disease (COVID-19):

– *“Coronavirus”*
– *“Corona_virus”*
– *“Corona-virus”*
– *“Novel_Coronavirus”*
– *“2019-nCoV”*
– *“Novel-Coronavirus”*
– *“NovelCoronavirus”*
– *“2019_nCoV”*
– *“nCoV”*
– *“COVID-19”*
– *“SARS-CoV-2”*
– *“covid19”*

At the end of this stage, we stored the collected data into our MySQL-Server, with data from each source in a different table. It should be remarked that the collected data are different in structure, and the ones from the fact-checking websites are labeled in various forms to describe real and misleading data. For example, the real data may be labeled as True, Real, Correct Attribution, Benar, Verdadeiro, Gerçek, Verdadero, etc., while the misleading ones could be labeled as False, Fake, Misleading, Falso, Faux, Engañoso, False Connection, False Context, False Content, C’est faux, etc. Hence, the data from different sources must be organized in a uniform format, and the labels need to be binarized to either Real or Misleading, as shown in the next stage.

Moreover, the published data in both the fact-checking websites and the official websites of international organizations are continuously increasing. Consequently, the amount of collected data is expected to change continuously. To build a near real-time detection system, we should continuously update our collected ground-truth to accommodate frequent updates from these organizations.

### B. Information-Filtering Stage

As we are interested in detecting misleading information that is written in English, the first step is to filter the collected data from different sources and select only written English data. The following steps are then carried out for standardizing our data and integrating them into a uniform ground-truth dataset.

#### 1) Duplicate Removal

We checked the collected data from the information-fusion stage and eliminated the redundant ones. This was done by removing the data that had the same content and originated from the same source, and keeping only one copy of them.

#### 2) Data Standardization and Label Biniarization:

We ensured the consistency of the data regardless of their source by making the data fit in a standard structure which contains the following fields:

– *Data_Publishing_Date (the date when the text was published from its source).*
– *Fact_Publishing_Date (the date when the text was checked and published on the fact-checking* websites).
– *Fact_text.*
– *Data_Origin (e.g., Facebook, Twitter, news website, blog, WHO, UNICEF, UN, etc.).*
– *Fact_publisher (e.g., politifact.com, snopes.com, factcheck.org, factual.afp.com, opensecrets.org, colombiacheck.com, truthorfiction.com, who.int, etc.).*
– *Label (e.g., Real* = 1*, Misleading* = 0*).*
– *Language (e.g., Arabic* = *’ar’, Spanish* = *’es’, French* = 0 *’fr’, etc. In the current implementation we are only interested in English* = *’en’).*

All date fields were reformatted to a standard date format (i.e., *YYYY-MM-DD*). Moreover, each fact was given a unique Fact_ID to be used for indexing purposes.

#### 3) Data Integration

After the data from different sources were indexed and standardized, they were inserted into the newly generated facts table.

### C. Model-Building Stage

**FIGURE 2.**
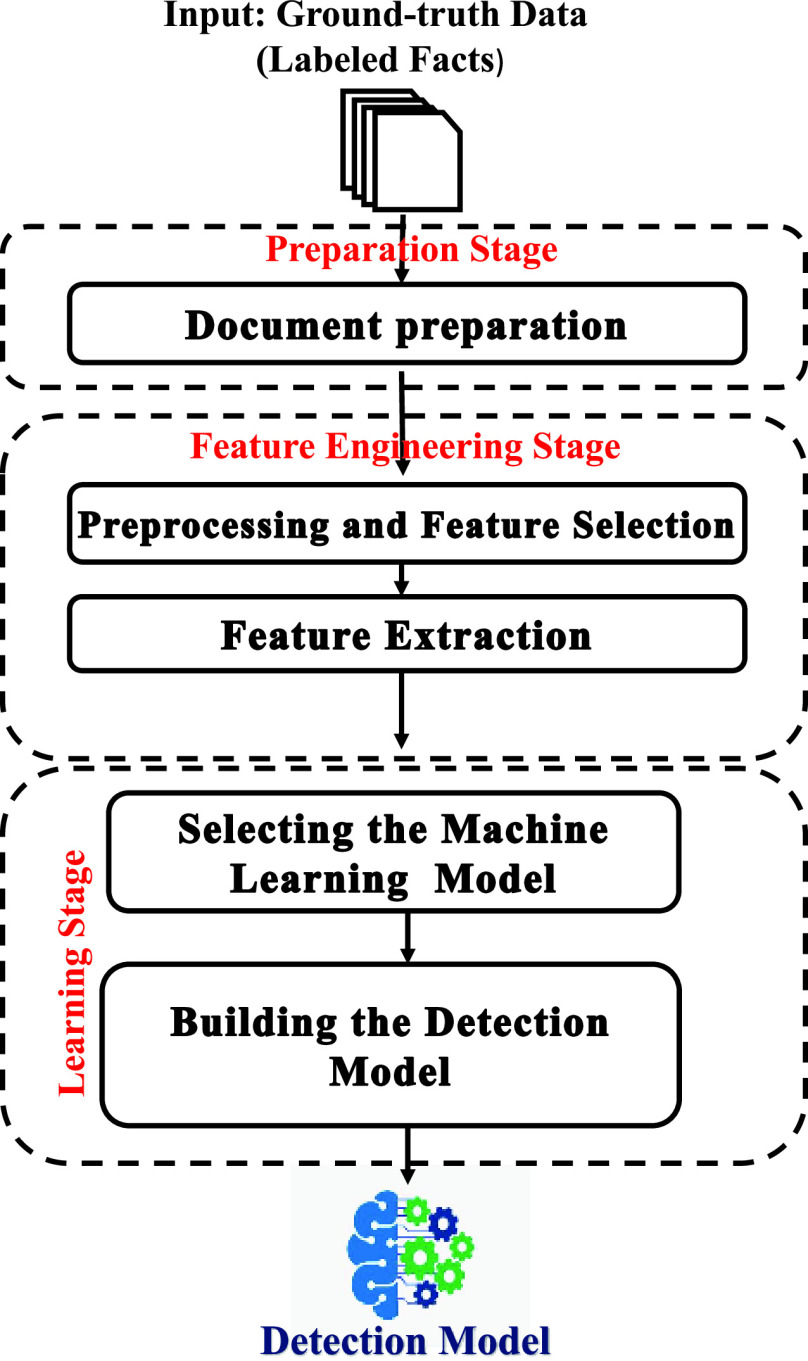
Block diagram of the detection model building stage.

To build the misleading-information detection model, all the collected ground-truth must be prepared first and then passed through Feature Engineering and Learning stages as shown in Fig. 2.

#### 1) Preparation

To build the detection model, the ground-truth data must be prepared first. This is done by utilizing the introduced technique in [16]. Each instance of the ground-truth data is represented by three fields: “Fact_Data”, “Label”, and “Language”. The “Fact_Data” field is obtained by the union of the original segments: Fact_text, Data_Publishing_Date, Fact_Publishing_Date, Data_Origin, and Fact_publisher. The “Label” field contains the label assigned to the ground truth instance, while the “Language” field indicates the language in which the instance is written.

#### 2) Feature Engineering

This stage is composed of two steps: a) Preprocessing and Feature Selection, and b) Feature Extraction as follows.

##### a: Preprocessing and Feature Selection [16]

This step aims to facilitate data manipulation, reduce memory space needed, and shorten the processing of huge amounts of data. This was done by extracting the Bag of Words (BoW) that represents the textual content of the collected data. Then, a hybrid set of features was selected from both the content of the collected data and its associated metadata.

The preprocessing was achieved by applying the following:

– Text Parsing: by detecting sentences and tokenizing the textual content of the collected data for further textual analysis.
– Data Cleaning: by applying regular expressions to keep only English alphabets, numbers, or any combination of them, and eliminating all symbolic and non-English alphabets. Also, all numbers in the Fact_Data in numeric written values were converted into textual written format (for example, “46” would be written as “Forty-six”) as introduced in [16].
– Part of Speech (PoS) Tagging: by marking-up each word in the text to a proper part of speech tag such as verb, noun, adjective, etc.
– Stop Words Removal: by removing the Stop Words. It was noted that it could reduce indexing size by as much as 20-30% [16].
– Stemming: by replacing each word by its corresponding root word to avoid redundant patterns. Porter English stemmer [46] was used to perform the word stemming process. It was noted that, by performing the stemming process, the indexing size could be reduced by as much as 40-50% [16].

Feature Selection was made by the following processes [16]:

– Applying capital letters heuristic to keep all words that begin with capital letters. As wherever there exists in the data a word that began with a capital letter, is an indication of its importance and it should not be neglected.
– Applying no-short heuristic to remove all words with the number of characters less than or equal to two.
– Considering only the words that were tagged as Verbs, Nouns, and Adjectives, to reduce the dimension of the extracted feature vector size, as these words are the most representative and descriptive parts in any textual data.
– Selecting relevant information from data, such as location-based, user-based, and time-based features. These metadata give a much more informative representation of the textual documents.
– Selecting the information related to the publisher and the source of data.

At the end of the Preprocessing and Feature Selection phase, we obtained a set of stemmed BoW which represents the original feature vector that would be used for the Feature Extraction phase [47], [48].

##### b: Feature Extraction

To the best of our knowledge from the literature, most researchers depend on the use of TF-IDF as a feature extraction technique. Hence, we used TF-IDF to give numerical weights for the textual content to be used for mining purposes. TF-IDF computes the importance of a term **t** based on how frequent **t** is within a document **d**, where it belongs, and its relative importance within the whole training dataset **D**.

The TF-IDF measure, as shown in equation (1), has a weight calculated by multiplying two values: the normalized Term Frequency (TF) and the Inverse Document Frequency (IDF) [49], [50].

\begin{equation*} \mathrm {TF-IDF}\left ({t,d }\right)\mathrm {= TF}(t,d\mathrm {) \times IDF(}t,D)\tag{1}\end{equation*}

#### 3) Learning

The extracted feature vectors, that represented each document in the training data, from the Feature Engineering stage were fed into different well-known classification algorithms. This is done by deploying the Scikit-learn Machine Learning library in Python [51]. First, we needed to validate the collected data. The ground-truth data were split into 80% training and 20% testing sets of 5-fold for cross-validation purposes. We used the training set to build detection models using different classification algorithms. As for the validation set, we passed it to the built detection models, and the obtained validation results are presented in subsection IV.C. Then, we used the whole collected ground-truth data as training data to build our misleading-information detection models using different machine learning algorithms.

### D. Detection Stage

To carry out the detection process, we used the detection models obtained in the Model-Building stage, to assemble an ensemble prediction model (Voting Ensemble). Then, we passed the query strings through the ensemble model and obtained the results of each model. Finally, we performed hard voting on all the results to get the detection decision. For example, suppose that we are using these 3 classification algorithms (Alg1, Alg2, and Alg3) and our data belong to two classes (Misleading and Real). We use the collected ground-truth data in building the detection models corresponding to each of Alg1, Alg2, and Alg3. Suppose that we need to predict the class of a query string (Q) as Real or Misleading. Assume that, after passing Q to these detection models, the resulting predictions from each model are as follows:

– Alg1 predicts class Misleading.
– Alg2 predicts class Real.
– Alg3 predicts class Real.

**FIGURE 3.**
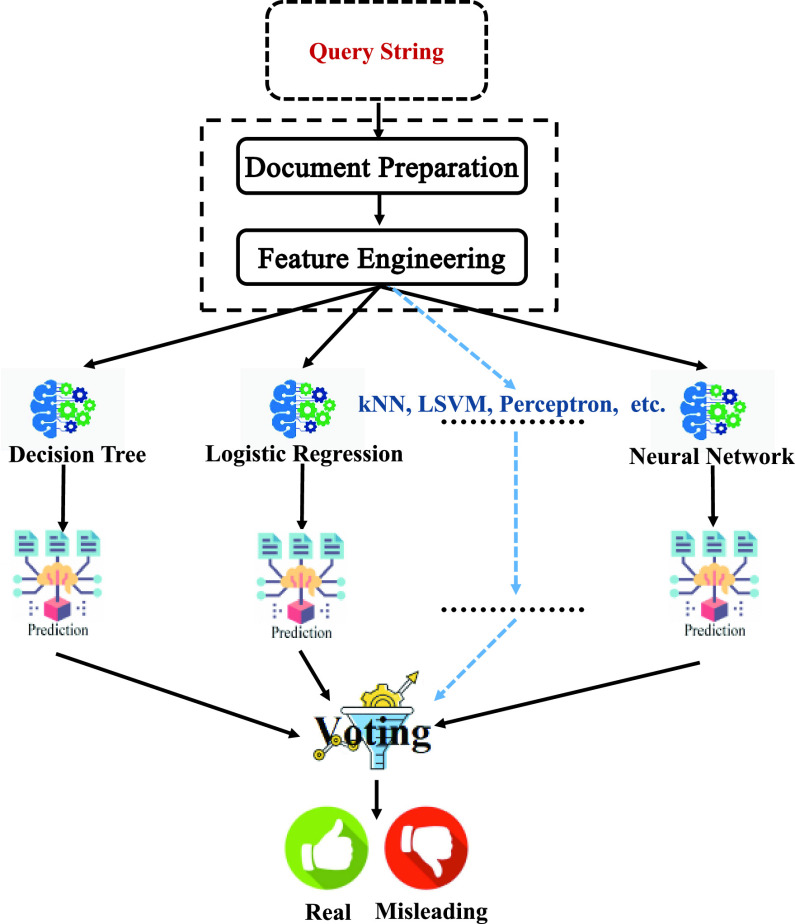
The voting ensemble method.

Two out of three classifiers predict class Real, so Real is the ensemble decision. Fig. 3 shows a diagram of the employed voting ensemble method.

**FIGURE 4.**
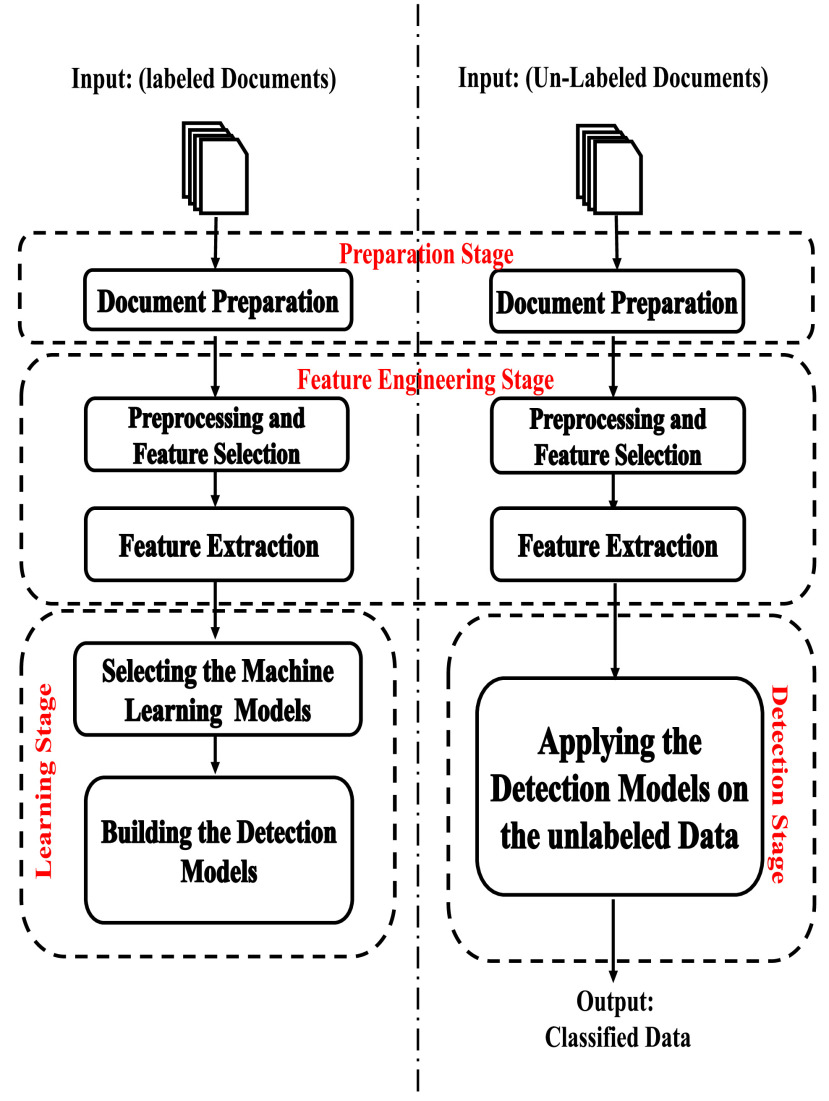
Block diagram of a misleading-information detection system.

For the query string to be classified as Real or Misleading, it must pass through the document Preparation and the Feature Engineering stages as previously discussed in subsection III.C.2. It is then submitted to the voting ensemble model for the class assignment process. To sum-up, Fig. 4 shows a block diagram of the process of building a misleading-information detection system, where the Model-Building part was discussed in III.C, and the Detection part was discussed in III.D.

## IV. Experimental Setup and Discussion

In this section, we discuss the experimental setup and the obtained performance evaluation of our models. We first performed validation on our collected ground-truth data. We used a 5-fold cross-validation technique with the ground-truth data randomly split into 80% training and 20% testing sets. Then, we built detection models using ten commonly used classification algorithms: Decision Tree (DT), k-Nearest Neighbor (kNN), Logistic Regression (LR), Linear Support Vector Machines (LSVM), Multinomial Naïve Bayes (MNB), Bernoulli Naïve Bayes (BNB), Perceptron, Neural Network (NN), Ensemble Random Forest (ERF), and Extreme Gradient Boosting classifiers (XGBoost). The models were built using the collected ground-truth data as described in subsection III.C.

### A. Ground-Truth Characteristics

The ground-truth data related to COVID-19 were collected through the WHO, UNICEF, and UN websites. These include all the textual data from speeches, reports, and news related to the COVID-19 outbreak before the WHO declared the pandemic on March 11, 2020 (from February 4, 2020 to March 10, 2020). Additionally, we deployed the Google Fact Check Tools API to collect available data from different fact-checking websites as discussed in subsection III.A.

The Google Fact Check Tools API helps users to easily search for facts online. For example, users can search for any keywords from a specific topic and obtain a list of matching claims and the corresponding facts. For querying the fact-checking websites in our current context, we used the search keywords as specified in subsection III.A, which we deemed as the most commonly used references related to the COVID-19 disease.

The collected data were stored in our MySQL-Server, and then we performed information filtering as described in subsection III.B. The resulted data from the Information-Filtering stage were labeled as Real or Misleading. The size of the collected ground-truth data is 7,486 instances. It should be remarked that the data that exist in both the fact-checking websites and the official international organizations are continuously changing and updated. Hence, the collected ground-truth should be updated regularly.

**FIGURE 5.**
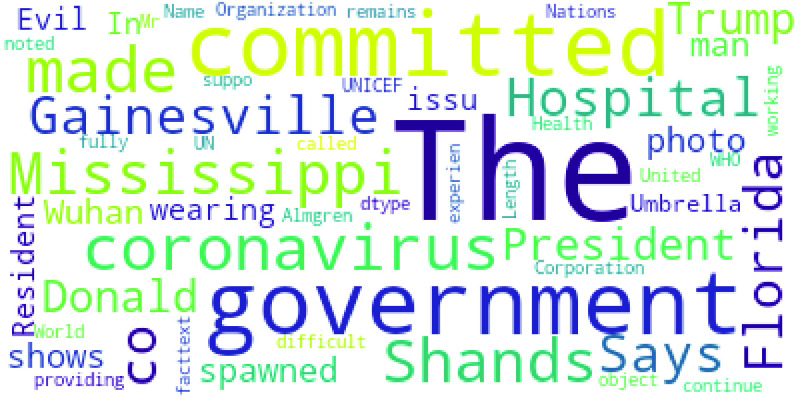
Word cloud.

**FIGURE 6.**
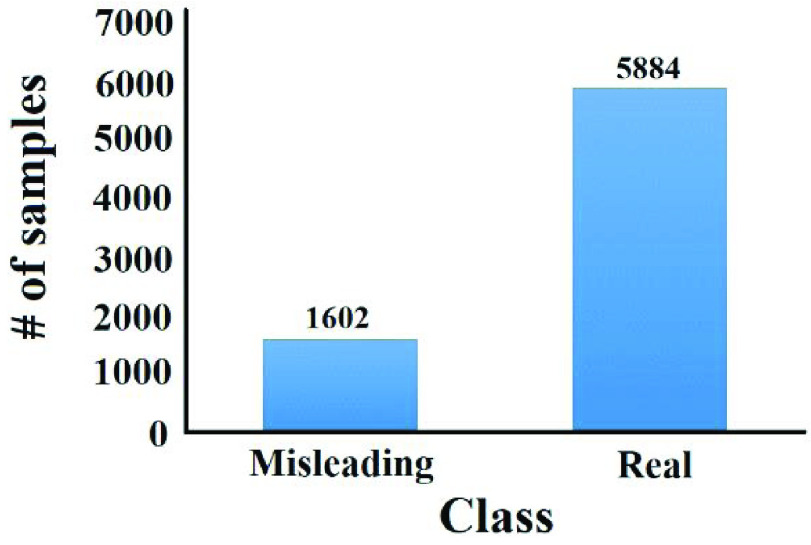
Distribution of ground-truth data.

We performed Exploratory Data Analysis (EDA) to get some general insights on them. Fig. 5 shows the word cloud of the top-100 words in the collected ground-truth data while Fig. 6 shows the distribution of ground-truth data classes.

**FIGURE 7.**
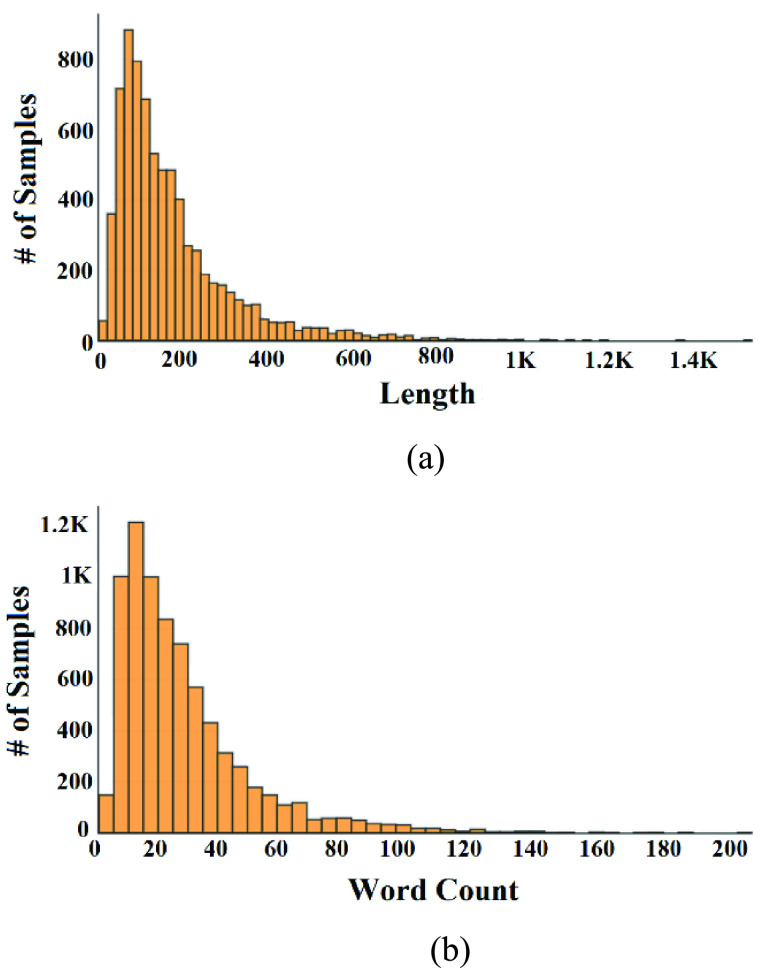
Distribution of ground-truth sample length and word count: (a) Samples’ length distribution, (b) Samples’ word count distribution.

Fig. 7 (a, b) show the distribution of length and word count of the ground-truth data.

**FIGURE 8.**
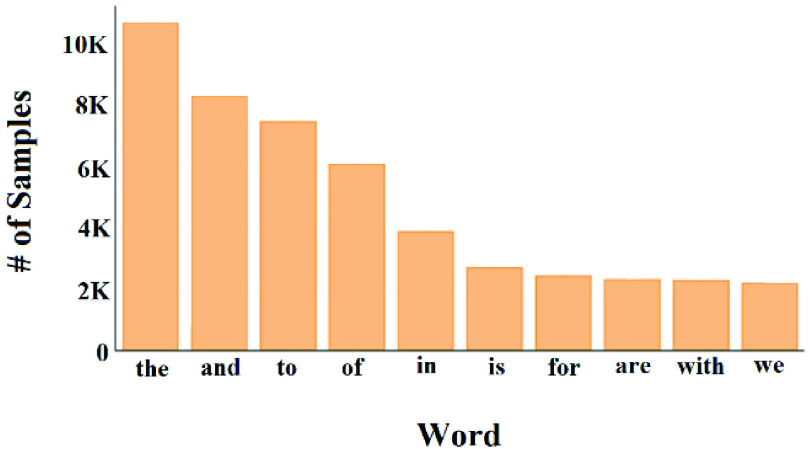
Distribution of top-10 unigrams.

We noticed from Fig. 7, that about 75% of the samples have less than or equal to 200 characters and less than or equal to 30 words. Fig. 8 shows the top-10 repeated unigrams in the ground-truth data.

From Fig. 8 we noticed that all the top-10 repeated unigrams in the ground-truth data are stop words and have relatively high frequencies. These stop words are useless when processing our data. This indicates that the data needs to be preprocessed to remove noisy and unimportant contents.

**FIGURE 9.**
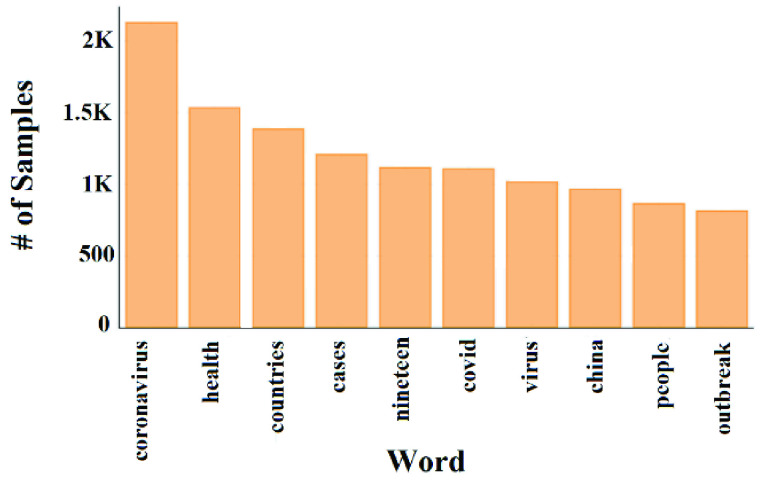
Distribution of top-10 unigrams after removing the stop words.

Fig. 9 shows the top-10 unigrams after removing the stop words. After performing the preparation and the preprocessing step, we were able to minimize the indexing size by around 75-80%.

**FIGURE 10.**
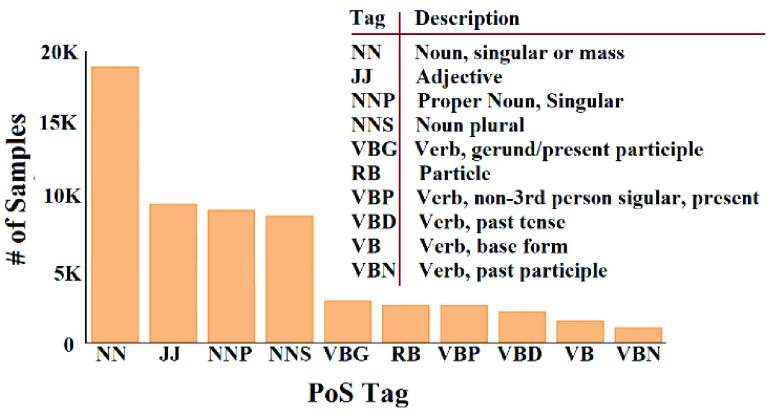
Distribution of top-10 PoS tags.

Fig. 10 shows the distribution of top-10 PoS tags and their description [52].

From Fig. 10 we noticed that the frequent words are mostly nouns, verbs, and adjectives. Hence, for dimension reduction of the extracted feature vector, we could consider only the words with these most frequent tags and neglecting the words with other tags.

### B. Evaluation Criteria

As the classification of a given document into either Real or Misleading is a binary classification problem, the evaluation of the classification results can be defined based on the confusion matrix [49], [53]. From the confusion matrix, 12 metrics are derived, as shown in Table 1, to evaluate the performance of the classifiers from various perspectives: Accuracy, Error Rate, Precision, Sensitivity, F1-Score, Specificity, Area Under the Curve, Geometric-Mean, Miss Rate, False Discovery Rate, False Omission Rate, and Fall-Out Rate [17], [54]–[58].

**TABLE 1:** Evaluation Metrics for Our Misleading-Information Detection System

#	Evaluation Metric	Formula	Focus
1	Accuracy (ACC)	$\frac {\mathrm {TP + TN}}{\mathrm {TP + TN + FP + FN}}$	It is the most used evaluation metric. It measures the ratio of correctly predicted instances over the total number of evaluated instances.
2	Error Rate (ERR)	$\frac {\mathrm {FP + FN}}{\mathrm {TP + TN + FP + FN}}$	It is the complement of Accuracy. It measures the misclassification error, which is the ratio of incorrectly predicted instances over the total number of evaluated instances.
3	Precision (P)	$\frac {\mathrm {TP}}{\mathrm {TP + FP}}$	It is used to measure the correctly predicted positive instances from the total predicted instances in a positive class.
4	Recall (R) or True Positive Rate (TPR) or Sensitivity (SN)	$\frac {\mathrm {TP}}{\mathrm {TP + FN}}$	It is used to measure the fraction of positive instances that are correctly classified.
5	F1-Score (F1)	$\frac {\mathrm {2 \times Precision \times Recall}}{\mathrm {Precision + Recall}}$	It is the harmonic mean of (P) and (R).
6	Area Under the Curve (AUC)	$\frac {1\mathrm {- FPR+TPR}}{2}$	It is used to construct an optimized learning model and for comparing learning algorithms. Its value reflects the overall ranking performance of a classifier.
7	Specificity (TNR)	$\frac {\mathrm {TN}}{\mathrm {TN + FP}}$	It is used to measure the fraction of negative instances that are correctly classified.
8	Geometric-Mean (GM)	$\sqrt {\left ({\mathrm {TNR\times TPR} }\right)}$	It is used to maximize the TPR and TNR while simultaneously keeping both rates relatively balanced.
9	Miss Rate (FNR)	$\frac {\mathrm {FN}}{\mathrm {FN + TP}}$	It is the proportion of positive instances that are incorrectly classified.
10	Fall-Out Rate (FPR)	$\frac {\mathrm {FP}}{\mathrm {FP + TN}}$	It represents the ratio between the incorrectly classified negative instances to the total number of negative instances.
11	False Discovery Rate (FDR)	$\frac {\mathrm {FP}}{\mathrm {TP + FP}}$	It represents the fraction of positive instances that are incorrectly classified as negative instances.
12	False Omission Rate (FOR)	$\frac {\mathrm {FN}}{\mathrm {FN + TN}}$	It is the proportion of false negatives that are incorrectly rejected.

In Table 1, TP (True Positive) and TN (True Negative) denote the number of positive and negative instances that are correctly classified, while FP (False Positive) and FN (False Negative) denote the number of misclassified positive and negative instances, respectively.

The experimental results from the built models were evaluated using all the metrics in Table 1. We wanted to be able to judge the performance of the various detection models from different perspectives and not depending only on a single viewpoint.

### C. Ground-Truth Validation Results

To test the validity of the ground-truth data, a 5-fold cross-validation technique was used with the ground-truth data randomly split into two sets (80% of the documents as a training set, and the rest is the testing set). Table 2 shows the obtained Accuracy, Error Rate and the Area Under the Curve of the obtained results from the ten classification algorithms (DT, MNB, BNB, LR, kNN, Perceptron, NN, LSVM, ERF, and XGBoost) when using TF and TF-IDF with (character level, Unigram, Bigram, Trigram, and N-gram word size), and word embedding as feature extraction techniques.

**TABLE 2:** Accuracy, Error Rate, and Area Under Curve of the Validation Results

			Classification Algorithm									
Metric	Feature Extraction		DT	MNB	BNB	LR	kNN	Perceptron	NN	LSVM	ERF	XGBoost
Accuracy (%)	TF		99.04	98.29	95.09	99.36	96.42	98.93	99.68	99.52	99.52	99.15
	TF-IDF	Unigram	98.93	98.01	93.96	98.18	98.67	99.57	99.63	99.52	99.15	99.25
		Bigram	98.02	97.92	90.55	97.49	97.06	98.72	98.99	98.99	98.56	96.80
		Trigram	93.00	92.57	92.68	92.47	90.92	93.54	93.75	93.91	93.43	91.45
		N-gram (n=2:3)	98.56	98.08	90.97	97.81	96.64	98.99	99.57	99.57	98.72	97.44
		Characters Level	99.20	97.97	91.35	98.08	99.36	99.52	99.63	99.63	99.09	99.41
	Word Embeddings		97.33	55.45	64.80	80.61	89.16	72.12	93.86	65.99	98.72	99.52
Error Rate (%)	TF		0.96	1.71	4.92	0.64	3.58	1.07	0.32	0.48	0.48	0.86
	TF-IDF	Unigram	1.07	1.99	6.04	1.82	1.34	0.43	0.37	0.48	0.86	0.75
		Bigram	1.98	2.08	9.46	2.51	2.94	1.28	1.02	1.02	1.44	3.21
		Trigram	7.00	7.43	7.32	7.53	9.08	6.46	6.25	6.09	6.57	8.55
		N-gram (n=2:3)	1.44	1.92	9.03	2.19	3.37	1.02	0.43	0.43	1.28	2.56
		Characters Level	0.80	2.03	8.65	1.92	0.64	0.48	0.37	0.37	0.91	0.59
	Word Embeddings		2.67	44.55	35.20	19.39	10.84	27.89	6.14	34.01	1.28	0.48
Area Under the Curve (%)	TF		97.91	96.87	90.58	98.74	93.45	98.83	99.41	99.03	99.03	98.33
	TF-IDF	Unigram	97.83	97.36	88.75	97.75	98.65	99.16	99.28	99.12	98.14	98.58
		Bigram	97.09	97.62	84.60	98.13	97.75	98.00	99.07	99.07	98.06	96.75
		Trigram	92.86	94.16	92.29	94.98	88.41	95.06	95.06	95.70	94.34	93.26
		N-gram (n=2:3)	97.15	97.67	84.66	98.43	96.91	97.96	99.34	99.34	97.68	97.24
		Characters Level	98.19	97.23	85.12	97.30	99.20	99.03	99.47	99.28	98.20	98.78
	Word Embeddings		94.68	63.05	65.02	70.54	82.48	60.20	89.46	57.59	97.85	99.21

From Table 2, the best ACC, ERR, and AUC evaluations are from the NN classifier, between 93.75% to 99.68%, 0.32% to 6.25%, and 89.46% to 99.47%, respectively. The ACC and the ERR measures, despite being easy to compute with less complexity, have limitations in the evaluation of a classifier and discrimination process.

One of the main limitations of ACC is that it produces less distinctive and less discriminable values. Consequently, its ability in selecting and determining the best classification algorithm is diminished. Besides, ACC is also less informative and biased towards minority class instances [55]. While for the AUC measure, it has been proven theoretically and empirically better than the ACC metric for evaluating a classifier’s performance and discriminating an optimal solution during classification training [59]. It should be remarked that although the performance of AUC is excellent for evaluation and discrimination, its computational cost is high especially when dealing with large datasets [55].

Table 3 shows single evaluation measures (either positive or negative class): The Precision, Recall/True Positive Rate/Sensitivity, and Specificity/True Negative Rate. In terms of measuring the positive patterns that are correctly predicted from the total predicted patterns in a positive class, and the fraction of negative patterns that are correctly classified, the best results are 99.93% and 99.74%, respectively, for the DT classifier. In terms of the fraction of positive patterns that are correctly classified, the best result is 99.87% when using the LR classification algorithm. Therefore, based on the evaluation results and what is the most important measure desired, a user can decide which classification algorithm to use for a specific purpose.

**TABLE 3:** The Precision, Sensitivity, and Specificity of the Validation Results

			Classification Algorithm									
Metric	Feature Extraction		DT	MNB	BNB	LR	kNN	Perceptron	NN	LSVM	ERF	XGBoost
Precision (%)	TF		99.86	99.32	99.36	99.80	98.77	99.00	99.87	99.87	99.87	99.73
	TF-IDF	Unigram	99.73	98.46	99.43	98.47	98.67	99.87	99.87	99.80	99.66	99.73
		Bigram	98.71	98.12	99.85	97.10	96.65	99.25	98.92	98.92	98.92	96.82
		Trigram	93.07	91.90	92.88	91.48	92.28	92.84	93.13	93.09	93.00	90.76
		N-gram (n=2:3)	99.59	98.34	99.70	97.45	96.48	99.73	99.73	99.73	99.46	97.56
		Characters Level	99.93	98.47	99.33	98.60	99.47	99.87	99.73	99.87	99.73	99.87
	Word Embeddings		99.45	95.68	94.73	81.85	95.80	84.87	97.51	85.63	99.33	99.73
Recall/True Positive Rate/Sensitivity (%)	TF		98.93	98.52	94.43	99.40	96.71	99.66	99.73	99.53	99.53	99.20
	TF-IDF	Unigram	98.99	99.06	92.95	99.26	99.66	99.60	99.66	99.60	99.26	99.33
		Bigram	98.78	99.25	88.13	99.80	99.73	99.12	99.80	99.80	99.25	99.19
		Trigram	98.44	99.32	98.24	99.73	96.54	99.46	99.39	99.66	99.12	99.25
		N-gram (n=2:3)	98.59	99.26	88.93	99.87	99.40	98.99	99.73	99.73	98.93	99.26
		Characters Level	99.06	98.99	89.73	98.99	99.73	99.53	99.80	99.66	99.13	99.40
	Word Embeddings		97.18	46.11	59.06	97.18	90.34	79.06	94.70	69.12	99.06	99.66
Specificity/True Negative Rate(%)	TF		99.48	97.38	97.64	99.22	95.29	96.07	99.48	99.48	99.48	98.95
	TF-IDF	Unigram	98.58	93.98	97.91	93.98	94.76	99.48	99.48	99.22	98.69	98.95
		Bigram	95.23	92.97	99.50	88.95	87.19	97.24	95.98	95.98	95.98	87.94
		Trigram	72.86	67.51	72.11	65.58	70.10	71.61	72.86	72.61	72.36	62.56
		N-gram (n=2:3)	98.43	93.46	98.95	89.79	85.86	98.95	98.95	98.95	97.91	90.31
		Characters Level	99.74	93.98	97.64	94.50	97.91	99.48	98.95	99.48	98.95	99.48
	Word Embeddings		97.91	91.89	87.17	15.97	84.56	45.03	90.58	53.40	97.38	98.95

Table 4 shows the F1-Score and Geometric-Mean validation results. The best results are 99.89% and 99.60% for both metrics F1-Score and Geometric-Mean when using the NN classifier. In general, these two metrics are considered as good discriminators and perform better than other metrics in optimizing classifiers, but only for binary classification problems and not for multiclass classification problems [58].

**TABLE 4:** The F1-Score and Geometric-Mean of the Validation Results

			Classification Algorithm									
Metric	Feature Extraction		DT	MNB	BNB	LR	kNN	Perceptron	NN	LSVM	ERF	XGBoost
F1-Score (%)	TF		99.39	98.92	96.83	99.60	97.73	99.33	99.80	99.70	99.70	99.46
	TF-IDF	Unigram	99.36	98.76	96.08	98.86	99.17	99.73	99.77	99.70	99.46	99.53
		Bigram	98.75	98.69	93.62	98.43	98.16	99.19	99.36	99.36	99.09	97.99
		Trigram	95.68	95.47	95.48	95.42	94.36	96.04	96.16	96.27	95.96	94.82
		N-gram (n=2:3)	99.09	98.80	94.01	98.64	97.92	99.36	99.73	99.73	99.19	98.40
		Characters Level	99.49	98.73	94.29	98.79	99.60	99.70	99.77	99.77	99.43	99.63
	Word Embeddings		98.30	62.23	72.76	88.86	92.99	81.86	96.08	76.50	99.19	99.70
Geometric-Mean (%)	TF		99.20	97.95	96.02	99.31	96.00	97.85	99.60	99.50	99.50	99.07
	TF-IDF	Unigram	98.79	96.48	95.40	96.58	97.18	99.54	99.57	99.41	98.98	99.14
		Bigram	96.99	96.06	93.64	94.21	93.25	98.17	97.87	97.87	97.60	93.39
		Trigram	84.69	81.88	84.17	80.87	82.27	84.39	85.10	85.07	84.69	78.80
		N-gram (n=2:3)	98.51	96.32	93.81	94.70	92.38	98.97	99.34	99.34	98.42	94.68
		Characters Level	99.40	96.45	93.60	96.72	98.82	99.50	99.38	99.57	99.04	99.44
	Word Embeddings		97.54	65.09	71.75	39.39	87.40	59.66	92.61	60.76	98.22	99.31

It should be remarked that Geometric-Mean aggregates both sensitivity and specificity measures for better discrimination between classes. As the objective of specificity usually conflicts with the objective of sensitivity, typically, the main goal of any classification algorithm is to improve the sensitivity, without sacrificing the specificity [58].

Finally, Table 5 shows different misclassification measures (Miss Rate, Fall-Out Rate, False Discovery Rate, and the False Omission Rate) for all the classification algorithms. These measures could help in choosing which algorithm to use in building a detection model. This choice is based on which measures that we want to keep as minimum as possible. For example, if we wanted to choose the detection model that had the lowest probability of false alarm (i.e., reducing the possibility of classifying a Real document as Misleading), we could choose the model that gives the lowest Fall-Out Rate. Whereas, if we wanted to reduce the rate of incorrectly classified Misleading documents as Real, we could choose the model that gives the lowest Miss Rate.

**TABLE 5:** The Miss Rate, Fall-Out Rate, False Discovery Rate, and False Omission Rate of the Validation Results

			Classification Algorithm									
Metric	Feature Extraction		DT	MNB	BNB	LR	kNN	Perceptron	NN	LSVM	ERF	XGBoost
Miss Rate (%)	TF		1.07	1.48	5.57	0.60	3.29	0.34	0.27	0.47	0.47	0.81
	TF-IDF	Unigram	1.01	0.95	7.05	0.74	0.34	0.40	0.34	0.40	0.74	0.67
		Bigram	1.22	0.75	11.87	0.20	0.27	0.88	0.20	0.20	0.75	0.81
		Trigram	1.56	0.68	1.76	0.27	3.46	0.54	0.61	0.34	0.88	0.75
		N-gram (n=2:3)	1.41	0.74	11.07	0.13	0.60	1.01	0.27	0.27	1.07	0.74
		Characters Level	0.94	1.01	10.27	1.01	0.27	0.47	0.20	0.34	0.87	0.60
	Word Embeddings		2.82	53.89	40.9	2.82	9.66	20.94	5.30	30.88	0.94	0.34
Fall-Out Rate (%)	TF		0.52	2.62	2.36	0.79	4.71	3.93	0.52	0.52	1.31	1.05
	TF-IDF	Unigram	1.42	6.02	2.09	6.02	5.24	0.52	0.52	0.79	1.31	1.05
		Bigram	4.77	7.04	0.50	11.06	12.81	2.76	4.02	4.02	4.02	12.06
		Trigram	27.14	32.49	27.89	34.42	29.90	28.39	27.14	27.39	27.64	37.44
		N-gram (n=2:3)	1.57	6.55	1.05	10.21	14.14	1.05	1.05	1.05	2.09	9.69
		Characters Level	0.26	6.02	2.36	5.50	2.09	0.52	1.05	0.52	1.05	0.52
	Word Embeddings		2.09	8.12	12.83	84.03	15.45	54.97	9.42	46.60	2.62	1.05
False Discovery Rate (%)	TF		0.14	0.68	0.64	0.20	1.23	1.00	0.13	0.14	0.34	0.27
	TF-IDF	Unigram	0.27	1.54	0.57	1.53	1.33	0.14	0.13	0.20	0.34	0.27
		Bigram	1.29	1.88	0.15	2.90	3.35	0.75	1.08	1.08	1.08	3.18
		Trigram	6.93	8.10	7.12	8.53	7.72	7.16	6.87	6.91	7.00	9.24
		N-gram (n=2:3)	0.41	1.66	0.30	2.55	3.52	0.27	0.27	0.27	0.54	2.44
		Characters Level	0.07	1.54	0.67	1.40	0.54	0.14	0.27	0.13	0.27	0.14
	Word Embeddings		0.55	4.32	5.27	18.15	4.20	15.13	2.49	14.37	0.67	0.27
False Omission Rate (%)	TF		4.04	5.58	18.20	2.32	11.86	1.34	1.04	1.81	2.84	3.08
	TF-IDF	Unigram	5.12	3.75	21.92	2.97	1.36	1.55	1.30	1.56	2.84	2.58
		Bigram	4.53	2.89	30.65	0.84	1.14	3.25	0.78	0.78	2.80	3.32
		Trigram	7.35	3.60	8.31	1.51	15.46	2.73	3.01	1.70	4.32	4.23
		ngram(n=2:3)	5.29	2.99	30.39	0.58	2.67	3.82	1.05	1.05	4.10	3.09
		Characters Level	3.54	4.01	29.09	3.99	1.06	1.81	0.79	1.30	3.33	2.31

In terms of Miss Rate, which represents the False Negative Rate (FNR), the best result is 0.13% when using the LR classifier. While in terms of the Fall-Out Rate, which represents the False Positive Rate (FPR) (also called False Alarm Rate (FAR)), the best result is 0.26% when using the DT classifier. It should be remarked that both the Miss Rate and the Fall-Out Rate are not sensitive to changes in data distributions and hence both metrics can be used with imbalanced data [58]. Additionally, the best obtained FDR is 0.07% when using the DT classifier, while the best obtained False Omission Rate result is 0.58% when using the LR classification algorithm.

From all the obtained results, it should be remarked that despite the NN, DT, and LR classifiers giving the best performance from different perspectives, all the results are satisfactory and indicate the validity of the collected ground-truth data. Hence, to get the benefits of different classification algorithms, we deploy the voting ensemble classifier in our detection model.

## V. Conclusion and Future Work

We proposed a novel framework that can be used for detecting misleading health-related information. It will help in detecting misleading information on any future global health issues, such as the anticipated second and third waves of Coronavirus, at the time of writing this article. If the topic of interest is not health-related, then the same framework can still be employed by changing the sources of information that are deemed unbiased and reliable, instead of the WHO, UNICEF, and UN.

In this article, we applied our methodology in detecting misleading information related to the COVID-19 outbreak. In this framework, we depended only on internationally reliable and independent institutions as sources of our ground-truth data to build the detection model with different detection algorithms. We scraped the WHO, UNICEF, and UN websites. These include all the textual data mentioned in speeches, reports, and published news related to the COVID-19 outbreak, before the declaration of the pandemic. Thus, the ground-truth dataset consists of information collected from February 4, 2020 to March 10, 2020.

Additionally, we deployed the Google Fact Check Tools API to collect available ground-truth from different fact-checking websites. We used these collected data in building a voting ensemble classifier for the detection task. Moreover, we performed a validation of the collected data to ensure their validity in building a detection system. We carried out 5-fold cross-validation on the data using ten classification algorithms and seven feature extraction techniques, and reported the results with twelve evaluation measures.

The results in general proved the validity of our collected ground-truth data and gave good insights into the performance of different classification algorithms on them. The best results are obtained from the NN, DT, and LR classifiers. The LR performs well with binary classification problems, and it could be considered as a one-layer NN. Additionally, we noticed that the results from the LR and the Perceptron are similar, as the LR is a Perceptron with a sigmoid function. For the final configuration of the detection system, it will depend on the classification algorithms that give the best results to build the ensemble detection model.

We deployed our proposed detection system in annotating 3,047,255 COVID-19 related tweets, and we made it publicly available to the research community (https://github.com/mohaddad/COVID-FAKES) [60].

There are several interesting future work directions:

1) Extend our proposed framework to include other trusted information sources such as the “International Committee of the Red Cross (ICRC)” [61].
2) Enrich the collected ground-truth data by including published information from the Twitter official accounts of the WHO [62], UNICEF [63], UN [64], and ICRC [65].
3) Enhance the web scraping process to eliminate ‘irrelevant’ data from the collected ground-truth data, for example, removing the contact-us information, the organization’s location, the descriptions that are associated with images, etc.
4) Extend the proposed framework to cover data written in other languages than English, to overcome the shortage of available multi-lingual detection systems, for example, cover the data written in Arabic, French, Spanish, Chinese, etc.
5) Use the proposed framework for detecting misleading information, shared or re-twitted on Twitter in a near real-time manner.
